# Unexpected high circulation of *Plasmodium vivax* in asymptomatic children from Kédougou, southeastern Senegal

**DOI:** 10.1186/s12936-017-2146-8

**Published:** 2017-12-29

**Authors:** Makhtar Niang, Fode Diop, Oulimata Niang, Bacary D. Sadio, Abdourahmane Sow, Ousmane Faye, Mawlouth Diallo, Amadou A. Sall, Ronald Perraut, Aissatou Toure-Balde

**Affiliations:** 1 Immunology Unit, Institut Pasteur de Dakar, Dakar, Senegal; 2 Arbovirus and Viral Hemorrhagic Fevers Unit, Institut Pasteur de Dakar, Dakar, Senegal; 3 West African Health Organization, Ouagadougou, Burkina Faso; 4 Medical Entomology Unit, Institut Pasteur de Dakar, Dakar, Senegal

**Keywords:** Malaria, *Plasmodium falciparum*, *Plasmodium vivax*, ELISA, IgG, MSP1, Asymptomatic carriage, Parasite circulation

## Abstract

**Background:**

Malaria in Senegal is due essentially to infections by *Plasmodium falciparum* and, to a lesser extent to *Plasmodium malariae* and *Plasmodium ovale*. By the use of molecular methods, detection of *Plasmodium vivax* has been recently reported in the region of Kedougou, raising the question of appraisal of its potential prevalence in this setting.

**Methods:**

A retrospective serological study was carried out using 188 samples taken from 2010 to 2011 in a longitudinal school survey during which 48 asymptomatic children (9–11 years) were recruited. Four collections of samples collected during two successive dry and rainy seasons were analysed for antibody responses to *P. vivax* and *P. falciparum*. Recombinant *P. falciparum* and *P. vivax* MSP1 antigens and total *P. falciparum* schizont lysate from African 07/03 strain (adapted to culture) were used for ELISA. Nested PCR amplification was used for molecular detection of *P. vivax*.

**Results:**

A surprising high prevalence of IgG responses against *P. vivax* MSP1 was evidenced with 53% of positive samples and 58% of the individuals that were found positive to this antigen. There was 77% of responders to *P. falciparum* outlined by 63% of positive samples. Prevalence of responders did not differ as function of seasons. Levels of antibodies to *P. falciparum* fluctuated with significant increasing between dry and rainy season (P < 0.05), contrary to responses to *P. vivax*. There was a significant reciprocal relationship (P < 10^−3^) between antibody responses to the different antigens, but with weak coefficient of correlation (Rho around 0.3) underlining a variable profile at the individual level. Clear molecular signature was found in positive IgG to *P. vivax msp1* samples by PCR.

**Conclusion:**

This cross-sectional longitudinal study highlights the unexpected high circulation of *P. vivax* in this endemic area. Sero-immunology and molecular methods are powerful additive tools to identify endemic sites where relevant control measures have to be settled and monitored.

## Background

Malaria remains a major threat in tropical and sub-tropical regions, with nearly 50% of the world population exposed to infective bites by Anopheles mosquitoes. Of the four species of *Plasmodium*, *Plasmodium falciparum* is responsible of 214 million cases in 2015 and almost half million deaths annually [1], essentially in sub-Saharan African children. Scaling up of integrated interventions strategies, including artemisinin-based combination therapy (ACT), universal coverage with long-lasting insecticide-impregnated bed nets (LLINs), systematic diagnosis using rapid tests (RDTs) has considerably reduced the burden of malaria in many countries. *Plasmodium vivax* has the widest geographic range and is responsible for 390 million clinical infections [2] along with a substantial mortality (1400–14,900 fatal cases) in endemic countries [1, 3]. *Plasmodium vivax* is the major cause of malaria outside of Africa, is responsible for long-term chronic illness and has dramatic consequences for global health and economy of endemic regions [4–6].

The two parasites coexist in large areas of the tropical and semi-tropical world, except, strikingly, in large parts of sub-Saharan Africa, where *P. vivax* appears to be almost completely absent [7, 8]. This situation is apparently caused by the high prevalence of the Duffy negative phenotype in the local populations, which was for a long time believed to confer complete protection against *P. vivax* malaria [9]. However, this belief is now being reconsidered upon several reports of *P. vivax* infections in Duffy negative individuals [10–15], as well as in countries where either *P. vivax* was absent, or was not detectable by the available methods as subsequently pointed by several reports for Eastern, Western and Central African countries [12–14, 16, 17].

PCR diagnosis is a highly sensitive method for detection of sub-microscopic circulating parasite species [18, 19]. However, some studies were negative when tracking of *P. vivax* in investigation of 2588 samples from West and Central Africa [20], and in Senegal by means of real time PCR and genotyping on 484 blood samples [21]. In contrast, recent studies reported molecular evidence of *P. vivax* infections in symptomatic patients in Senegal [22], in Mali [15] and in Cameroon [23]. The recent positive results from Kedougou region [22] led to enlarge the tracking procedure by investigating *P. vivax* antibody signature in a retrospective collection of sera selected from a long-term surveillance procedure settled in this geographical area.

In a first step, a screening was performed in a longitudinal survey involving a cohort of 48 asymptomatic school children and investigated for the potential co-circulation of *P. vivax* and *P. falciparum*. To this end, IgG responses to recombinant MSP1 antigens from the two species and whole parasite extract from *P. falciparum* were measured by ELISA. The specific presence of circulating *P. vivax* parasites in IgG positive samples was also investigated by molecular methods.

## Methods

### Study area, design and population

The study was conducted in the Kedougou region located in southeast Senegal bordering with Guinea, Mali and Gambia with the objective of investigating arboviruses infections and circulation. Details of the study area such as follow-up of population, climate, rainfall, landscape and fauna, have been previously reported [22, 24, 25]. Malaria investigation was an integral part of the project as a major cause of fever in that area leading to similar symptoms to those from virus infections requiring differential diagnosis. In this region, malaria remains highly prevalent with an incidence > 25‰ of confirmed clinical malaria cases. In Kedougou, transmission is high and seasonal. National Malaria Control Programme reported in 2013–2014 a total of 37,053 confirmed clinical cases including 1012 severe case, resulting in a prevalence of 255.5‰ [26].

The project recruited 48 school children from the Catholic Mission School in Kedougou (28 M/20 F, aged 8–11 years in May 2010) that were followed-up during 2 consecutive years (2010 and 2011) and sampled twice each year during the dry and rainy seasons (May and December, respectively). Children were asymptomatic for malaria episode at the recruitments.

### Antigens and ELISA procedure

Recombinant MSP1 antigens from *P. falciparum* and *P. vivax* were secreted following infection of *Trichoplusia* in insect cells (High Five, Invitrogen) with recombinant baculovirus, and purified by chromatography using Talon metal affinity resin, as previously described [27, 28]. The baculovirus expression system has been shown to ensure optimal reproduction of conformational epitopes including EGF domains [29]. The whole parasite extract antigen was from the 07/03 Dielmo strain adapted to in vitro culture. Preparation and use of the schizont extract (SE) was done as described previously [30–32]. Antigens were diluted in PBS and used to coat Immulon-4 plates at a concentration of 1 µg mL^−1^ for MSPs and diluted at 1:320 for SE after calibration [31].

Levels and prevalence of IgG responses were quantified by ELISA with duplicate sera samples diluted 1:200 using standard procedure already described [33–35]. Positive and negative controls were included in each assay i.e. a pool of 25 sera from clinically immune adults living in the village of Dielmo (a holoendemic area of malaria transmission in Senegal), a pool of 20 sera from *P. vivax* infected patients from Madagascar (kind gift from Dr Inès Vigan-Womas) and a pool of European and/or African non-immune sera, respectively. Results were expressed as OD ratio = OD sample/OD naive serum pool. Sera showing an OD ratio > 2 (corresponding to the signal of naive controls + 3 SD) were considered sero-positive for prevalence analysis.

### Molecular detection of *Plasmodium* species

The qualitative detection and characterization of *Plasmodium* spp. were performed on a subset of *P. vivax* serology-positive and negative samples selected from the four sampling periods and from five children using nested PCR amplification according to the method described by Snounou et al. [19]. Genomic DNA (gDNA) isolated from frozen serum samples as described previously [18, 36] were purified and concentrated using Genomic DNA clean and concentrator™^-5^ (catalogue number D4014, Zymo Research, Irvine, USA) according to manufacturer’s instructions. For the first round of PCR amplification, *Plasmodium* genus-specific primers (rPLU6 and rPLU5) were used for amplification of the *18S small subunit ribosomal RNA* (*18S ssrRNA*) genes. The nested amplification was performed using species-specific primers rVIV1 and rVIV2 to amplify a *P. vivax*-specific 120 bp fragment of *ssrRNA genes*. DNA from blood samples of confirmed *P. falciparum*, *P. malariae*, *P. ovale* and *P. vivax* infected patients served for positive controls in all amplifications [22].

### Statistical analysis

Antibody levels and prevalence of responders in different groups were compared using the Mann-Withney and Wilcoxon signed rank test, the Spearman rank correlation test for non-normally distributed paired data and the fisher exact test. P values < 0.05 were considered significant. Statistical analyses were performed with R and Statview 5.0 (SAS Institute) software.

## Results

### Prevalence and levels of antibody responses against antigens tested

**Fig. 1:**
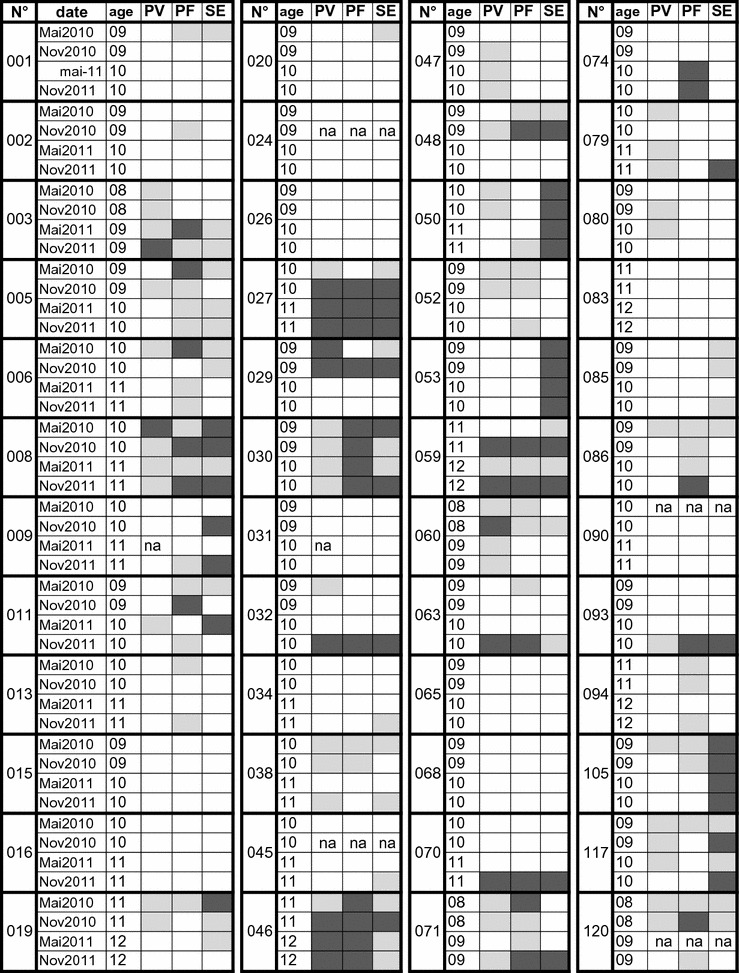
Detailed schematic results of individual responses of children to PvMSP1, PfMSP1p19 and SE during the 2 years’ follow-up. This graph summarizes antibody responses measured during the follow-up for each individual. IgG responses against PvMSP (PV), PfMSPp19 (PF) and Schizont extract (SE) were stratified as function of the magnitude of responses i.e. negative (white square), positive (ODratio > 2 and < 5, light grey square) and strong (ODratio > 5, dark grey square). Missing samples are labeled na

Results of prevalence of responders and mean levels of antibody responses against PfMSP1p19, PvMSP1 and SE for each set of samples from 2010 and 2011 are summarized in Table 1. A surprising high prevalence of antibodies to *P. vivax* was revealed in this survey with 53% of positivity of overall samples tested and a median level of 1.5 OD ratio. When prevalence was calculated as function of the number of children with at least one positive sample against *P. vivax* MSP1 antigen during the survey, only 20 children remained negative and 28 positive i.e. 58% of responders (Fig. 1).

**Table 1:** Prevalence and mean antibody levels measured in sequential samples from 48 children in Kedougou years 2010 and 2011

Antigen tested	May 2010	November 2010	May 2011	November 2011	Overall
No individuals	No = 47	N = 46	N = 47	N = 48	N = 188
Ag MSP1					
Mean OD [min–max]	0.57 [0–1.93]	0.62 [0–1.93]	0.47 [0–1.36]	0.82 [0–2.25]	0.52 [0–2.25]
*P. vivax*					
Mean OD ratio [min–max]	2.6 [1–7.1]	2.9 [1–6.4]	2.2 [1–4.5]	3.7 [1–7.2]	2.9 [1–7.2]
No positive (prevalence)	40%	41%	25%	31%	53%
Pf MSP1p19					
Mean OD [min–max]	0.11 [0–0.98]	0.21 [0–2.10]	0.10 [0–1.19]	0.30 [0–2.14]	0.18 [0–2.14]
Mean OD ratio [min–max]	2.5 [1–16.7]	4.0 [1–34.7]	2.3 [1–20.0]	5.4 [1–33.8]	3.5 [1–33.8]
Prevalence	43%	38%	23%	46%	61%
Ag SE 07_03					
Mean OD [min–max]	0.58 [0–2.45]	0.65 [0–2.41]	0.48 [0–2.39]	0.82 [0–2.59]	0.63 [0–2.59]
Mean OD ratio [min–max]	2.5 [1–10.5]	2.8 [1–10.4]	2.2 [1–9.6]	3.5 [1–12.3]	2.8 [1–12.3]
No positive (prevalence)	45%	37%	28%	46%	63%

Regarding *P. falciparum*, prevalence and levels of antibody responses were high with 61 and 63% of positive samples against PfMSP1p19 and SE, respectively and a median OD ratio of 1.6 for the two antigens. When calculating prevalence for the actual number of individuals having a positive sample, 37 children were found positive at least once during the survey i.e. 77% of responders in the survey.

The antigen-specific longitudinal fluctuations of prevalence between the 4 monthly collections of samples were not significant (Table 1, Fisher exact test), except one significant increase of responders against PfMSP1p19 between May (23%) and November (46%) 2011.

### Distribution of IgG responses against *P. vivax* and *P. falciparum* in individuals followed-up in 2010–2011

Longitudinal analysis of antibody responses measured during the follow-up is detailed for each individual on Fig. 1. Antibody responses were stratified as negative (white), positive (grey) and elevated (OD ratio > 5; dark grey) and illustrated as a function of time for each child. Profiles of antibody responses appeared substantially variable among individuals: some children remained completely antibody negative during the 2-years survey against the two species (n = 9), and some others showed low (n = 10) or high (n = 3) antibody responses to one parasite species (*P. vivax* or *P. falciparum*). Of note, 10 children showed antibodies to *P. falciparum* but not to *P. vivax* and two children had antibodies to *P. vivax* alone.

### Longitudinal comparison of levels of Ab responses

**Fig. 2:**
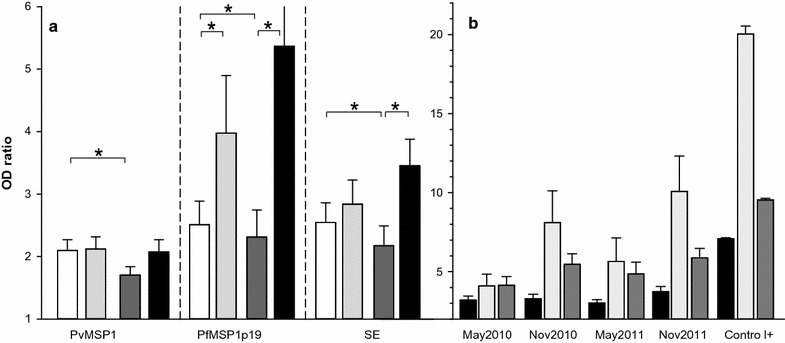
Levels of IgG responses to *P. falciparum* and *P. vivax* as function of time of follow-up. Longitudinal fluctuation of antibody levels is plotted in part **a** as histogram + SE for each Ag in May 2010 (white), November 2010 (light grey), May 2011 (dark grey), November 2011 (black). Asterisks indicate significant different level of Ab (P < 0.05, Wilcoxon signed rank test). In panel **b**, antibody levels for all positive responders (individuals with ODratio > 2) to PvMSP1 (black), PfMSP1p19 (light grey), SE (dark grey) are shown as histogram plots + SE for each set of samples from May 2010 to November 2011. IgG levels of positive controls are also shown for comparison

Mean levels of IgG responses measured in the four sets of samples are plotted on Fig. 2a. The magnitude of antibody responses from positive individuals with ODratio > 2 is illustrated on Fig. 2b. Antibody responses to *P. vivax* MSP1 fluctuated moderately with a slight significant decrease (P = 0.045, Wilcoxon test) when comparing dry seasons of May 2010 vs May 2011. On the contrary, antibody responses to *P. falciparum* showed some substantial fluctuations. Antibodies to PfMSP1p19 significantly increased with the rainy seasons in 2010 and 2011 compared to the dry season. In addition, a significant lower level of responses was observed in May 2011 compared to May 2010 (P < 0.05). For the SE antigen, antibody profile of response was almost similar to PfMSP1p19, underlining a significant increase during the rainy season in 2011 and a lower level in May 2011 compared to May 2010.

Regarding magnitude of antibody responses from high responders, mean levels were quite high (almost 50% of the levels of positive controls). Importantly, some individuals showed strong responses (Fig. 2b) with higher antibody levels than positive immune controls i.e. PvMSP1 (n = 2), PfMSP1p19 (n = 6) and SE (n = 7).

### Relationship between antibody responses to the different antigens

**Fig. 3:**
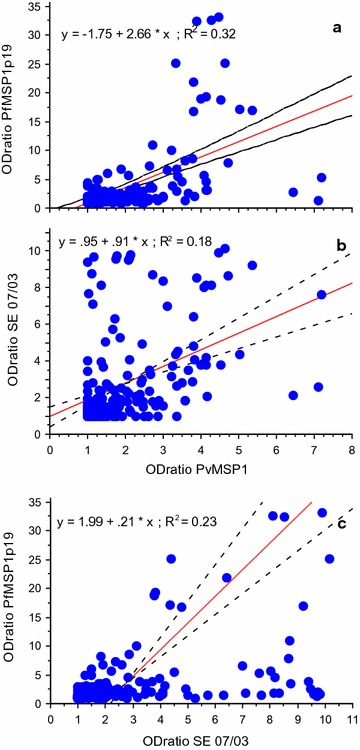
Correlation between IgG responses to the different antigens tested. Relationship between anti-PvMSP1 IgG responses and Ab to PfMSP1p19 and SE are plotted in **a** and **b**. Relationship between IgG responses to PfMSP1p19 and SE are plotted in **c**. Linear regression curve with 95% confidence interval are also plotted including the equation of the respective curve and their coefficient of correlation

The reciprocal correlation between levels of IgG against PvMSP1, PfMSP1p19 and SE was analysed by Spearman rank test and illustrated as XY plot including a linear regression curve on Fig. 3. There was a significant (P < 10^−3^) inter-relationship between IgG responses to PvMSP1 *vs* PfMSP1p19 (Rho = 0.56), PvMSP1 vs SE (Rho = 0.49), PvMSP1 vs FfMSP1p19 (Rho = 0.62). As shown on Fig. 3, correlation coefficients were weak from 18 to 32% in all cases. When analyzing by means of three level contingency tables (negative–positive–strong) it appears a score of 35–37% of discrepancy in responses.

Taken together with observations shown on Fig. 1, there is a variable profile of antibody response against *P. vivax* and *P. falciparum* at the individual level, outlining a relatively independent (non linear) profile of recognition of the three antigens in such a semi-longitudinal follow-up.

### Molecular characterization of the *P. vivax* parasite in positive individuals

For the confirmation of presence of circulating *P. vivax* parasite in children, nested PCR testing was performed in a subset of samples that were positive for IgG to *P. vivax* Msp1 antigen to which were added as control samples from five negative individuals from the same cohort.

**Fig. 4:**
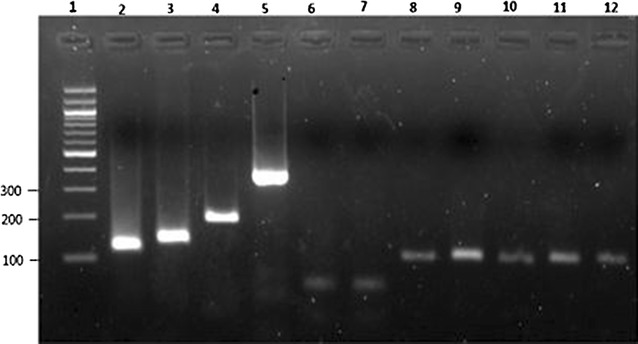
Gel picture showing detection of *Plasmodium* 18S ssrRNA gene. Lane 1 was loaded with 100 bp DNA Ladder. Positive amplification of *P. vivax* (120 bp), *P. malariae* (144 bp), *P. falciparum* (205 bp), and *P. vivax* (120 bp) is shown on lanes 2–5, respectively. Positive amplification of *P. vivax* 18S ssrRNA gene in serology-positive samples to PvMSP1 from five children 008, 027, 046, 032 and 059 (lanes 8–12) and the four sampling periods [May 2010 (lane 8), November 2010 (lanes 9 and 12), May 2011 (lane 10) and November 2011 (lane 11)] is also shown. Lack of amplification of *P. vivax* serology negative samples 015 (May 2010) and 016 (November 2010) is also shown on lanes 6 and 7, respectively

Results shown on Fig. 4 revealed the presence of amplified DNA in parasite control samples corresponding to *P. falciparum* (205 bp), *P. vivax* (120 bp), *P. malariae* (144 bp) and *P. ovale* (375 bp). Importantly, *P. vivax* 18S ssrRNA gene was also amplified in all selected serology-positive samples, as illustrated in Fig. 4 for samples 8–12 corresponding respectively to samples 008 (May 2010), 027 (November 2010), 046 (May 2011), 032 (November 2011) and 059 (November 2010) and originating from the four survey periods while no amplification was obtained for serology-negative samples as illustrated for the two samples shown in the gel that correspond to samples 015 and 016 from May 2010 and November 2010, respectively.

## Discussion

Several recent findings have changed the vision of transmission of *P. vivax* in Africa with evidence of much more important presence of this parasite in West Africa. The dogma of a protecting barrier against *P. vivax* infection due to widespread Duffy-negativity in African population [9] has been revisited. There were already observations arguing for the presence of the parasite as nonimmune travelers returning from Central or Western Africa showed clinical infections diagnosed as being due to *P. vivax* that persisted over years of surveillance [37]. Furthermore, an increasing number of reports highlighted the capacity of *P. vivax* parasite to invade and infect Duffy negative individuals firstly in endemic areas, such Brazil [38], Ethiopia [39], Madagascar [11], Mauritania [14], but also in West African countries including Cameroon [10, 40], Equatorial Guinea, Angola [12] and recently Mali [15]. Thus, the real burden of *P. vivax* in Africa is largely unknown. The partial view of *P. vivax* epidemiology in Africa is due to several reasons starting with the dogma of Duffy-negative resistance to P. vivax infection, the low level of *P. vivax* parasitaemia compared to *P. falciparum* hardly detectable on slides, and limited tools available in different laboratories for affordable large-scale screenings. In addition, both relevant methods such as PCR and microscopy may not detect previously-cured *P. vivax* infections. For these reasons, a species- specific ELISA assay represents a complementary tool for population-based screening surveys in the absence of affordable rapid field test, comparable to the largely distributed ones for *P. falciparum* field detection.

Several studies underline antibodies to merozoite antigens as valuable biomarkers of immunity [41, 42]. Therefore, among these, MSP1 antigens from *P. falciparum* and *P. vivax* are interesting targets as biomarkers of exposure and protection. PfMSP1 has been used for longitudinal retrospective sero-surveillance in the Gambia providing relevant information about population immunity as well as exposure, in the context of declining transmission and immunity [43]. Importantly, MSP1 antigens from *P. falciparum*, *P. vivax*, *P. malariae*, *P. ovale* were shown to clearly indicate proportional exposure to these species without any cross-reactivity in Zimbabwean population [44]. The baculovirus expressed recombinant MSP1 antigens used in this study are highly recognized antigens harbouring conformational epitopes including EGF domains [45, 46]. They have been reported as relevant vaccine candidates [34, 47], including effective protection in experimental primate model for PvMSP1 [48]. Both MSP1s have been recently used as biomarkers in ELISA multiplex studies in cohorts from Cambodia, Senegal and Côte d’Ivoire [49–51].

Recent studies used serological testing with recombinant *P. vivax* circumsporozoite protein (PvCSP) and PvMSP1. Both of them are expressed in liver-stage parasite and post-hepatic merozoite even without blood infection and led to substantial levels of prevalence i.e. 13% in Congo by passive case detection [52] and almost 30% in Beninese blood donors [53]. Therefore, PvCSP and PvMSP1 showed high sensitivity and > 96% specificity highlighting the best predictive positive value of 93.3% for PvMSP1 [53]. Compared to single target antigens, whole parasite extracts as SE from *P. falciparum* used here has the advantage of containing a broad panel of parasite antigens, which moreover are presented in their native conformation. Well-defined SE antigen also appears as relevant antigen tool for monitoring fluctuation of anti-parasite antibody responses as function of transmission [33, 50, 51, 54], however whole parasite extracts are not available for *P. vivax*, a parasite presently not adapted to long-term in vitro culture.

An important point of this study relies upon the use of school surveys as such cohorts are considered as an appropriate target population somewhat easily accessible. In areas with decreasing transmission, the malaria risk become higher in 5–14 years old children than in the under 5 years old [55]. Therefore, measures of seroprevalence in this age group is very useful for estimating short-term changes in the burden of infection over a broad location. Thus, school-based surveys were revealed as an effective and highly relevant alternative to population-based surveys for identifying potential foci of transmission in areas with varying and/or decreasing transmission [56]. Recently, a pilot survey involving 32 schools in The Gambia identified potential hot spots in a large area of heterogeneous transmission [57]. Here the investigation in a longitudinal manner increased the detection capacity and resulted in a surprising high level of prevalence of IgG responses against *P. vivax*. The individual prevalence was 58% high i.e. almost twice higher than the result from single transversal measure from the dry season of May 2011. Such level of prevalence is also substantially higher than in Beninese blood donors 28.7% [53], than in Congo clinical cases 13% [52] or than in Cameroon symptomatic, asymptomatic adults (38, 15%) [10, 23]. All these reports were from cross-sectional studies, it is possible that longitudinal studies in those settings could reveal much higher level of circulation of *P. vivax*. Importantly, screening of schoolchildren in the present study raises the question of the considerable heterogeneity in the geographic distribution of transmission intensity in Senegal as function of climatic variation from north to south, and environmental factors including human activities [26]. In Kedougou, there is a relatively high malaria transmission as indicated by an EIR of 100–200 so that population live in co-endemic area with active transmission of *P. falciparum*, *P. ovale* spp. and *P. malariae* [21]. Strikingly, 9 out of 48 children (19%) remained completely negative during the 2 years of follow-up. Such observation raises the question of existing potential “hot/cold” spots of transmission requiring further investigation. As a matter of fact, living in co-endemic areas with active transmission of *P. falciparum*, *P. ovale* spp. and *P. malariae* was predictive of that exposure to infection by one malaria species would also be predictive of the risk as to exposure to another malaria species [52]. Likewise, responders to *P. vivax* were also responders to *P. falciparum* (28 out of 30), an observation supporting the hypothesis that co-circulation is a favorable environment for higher risk of *P. vivax* circulation. Indeed, co circulation is related to active transmission by mosquitoes as found in *Anopheles gambiae* and *Anopheles funestus* [13]. Further investigations are required to address these questions in Kedougou region; a pertinent survey should involve multispecies specific ELISA assay and PCR as complementary tools for malaria surveillance [55].

The presence of circulating *P. vivax* parasites revealed by nested PCR in a subset of serology-positive individuals tested suggests active *P. vivax* infections in this asymptomatic cohort with no clinical signs and symptoms of malaria at the time of sampling. *P. vivax* malaria has an atypically long incubation period in a large proportion of individuals but a typically mild clinical course [58]. Together, the recent [22] and current reports of *P. vivax* infections respectively in symptomatic and asymptomatic individuals are suggestive of an important circulation of *P. vivax* parasites in Kedougou. The parallel findings between the PCR positivity and sero-reactivity are in line with studies performed by others groups [49, 59] that observed tight correlations as well.

## Conclusion

Taken together, these findings strongly suggest a high level of *P. vivax* transmission and the ability of the parasite to invade red blood cells and to persist and potentially expand through the human-mosquito transmission cycle. Several complementary investigations on these points are required and the true prevalence of *P. vivax* in Senegal should be questioned by investigation in other regions.
